# Best Distance Perception in Virtual Audiovisual Environment

**DOI:** 10.1155/2022/6010667

**Published:** 2022-06-28

**Authors:** Hui Song, Ke Ma

**Affiliations:** ^1^Art College, Northeast Electric Power University, Jilin, China; ^2^Jilin Ultrahigh-voltage Company of Jilin Electric Power Co. Ltd. of STATE GRID Corporation of China, Jilin, China

## Abstract

Auditory distance perception is important to virtual sound production and is affected by many factors. In order to investigate the effect of the visual cue on auditory distance perception and the best auditory distance perception in the existence of virtual sound source, experiments on the effect of visual cue on distance perception and the best auditory distance perception were conducted, respectively. The results of the first experiment showed that there was no obvious difference between the auditory distance perception in the existence and absence of virtual sound source, but visual cue can decrease the fluctuation of the perception. From the best auditory distance perception experiment, the attenuating SPL of the initial sound signal that made the subjects perceive the best auditory distance in the existence of virtual sound source at the distance of 4m, 6m, 8m, 10m, and 12m was measured, respectively, and compared the difference of attenuating SPL between experiment measurement and theoretical calculation.

## 1. Introduction

Virtual reality technique has been applied in audio and video production widely, such as games, films, and animations [1, 2]. In terms of image production, computer graphics and image processing techniques provide mutual technical support to the visual perception and visual interaction [3, 4], and in terms of sound production, sound field simulation provides technical support on sound reproduction and generation [5, 6]. But the developments of the two aspects are relatively independent. There are no inner and organic relationships between image production and sound production. They are always mutually independent and lack the interaction between visual information and subjective listening perception. As we all know, sound perception depends on visual information [7], and the expression and information that sound delivered must be consistent with the visual information. They can complement and reinforce each other [8]. A good sound effect can help the audience to understand the play's plot or the scene and immerse in the play. If the sound effect does not match the image on the screen, the film will lose its completeness and the harmony of subjective perception.

In order to obtain the same spatial listening perception with the natural environment, sound-processing technology on recording, production, and replay has achieved a great progress [9], and the concept of virtual sound environment has been proposed. Virtual sound environment includes two aspects, the narrowly defined concept and broad concept [10].

The narrowly defined concept of virtual sound environment mainly emphasizes the physical process of simulating sound signal generating, transmitting, and reception, which is also called auralization, and the sound signal is also called the realistic sound or the 3D sound [11].

The broad concept of virtual sound environment has two meanings. One is the exact reproduction of the real sound scenes, and the other one is simulating the sound field effect which matches the picture content by sound field simulation method for the virtual scenes in the screen [12]. How to produce sound that matches the scenes is becoming a scholarly research focus.

As the application of virtual reality is becoming more and more widely used in many fields, sound reproduction is receiving more and more attention. Auditory distance perception is a crucial factor in sound reproduction. Research on distance perception is important to sound recording and sound processing technology in the virtual sound environment. The present work is aimed to investigate the optimal signal processing method of auditory distance simulation by psychoacoustics experiment.

This article includes 6 parts:

1. Introduction: introduced the necessity of research on distance perception.
2. Factors that Affect Sound Distance Perception: This part reviewed in detail the research status and development of the factors that affect sound distance perception.
3. Comparison of Auditory Distance Perception in the Existence and Absence of Visual Cue: this part introduced serial experiments of sound distance perception in the existence and absence of visual cue to investigate the effect of the visual cue on sound distance perception.
4. Experiment of Best Distance Perception: this part introduced serial experiments of best distance perception in the existence of virtual sound source at a distance of 4m, 6m, 8m, 10m, and 12m.
5. Discussion: this part analyzed the difference between the theoretical calculation and experimental measurement of SPL attenuation when the virtual sound source is at a distance of 4m, 6m, 8m, 10m, and 12m.
6. Conclusions, Limitations, and Future Work: this part concludes the work of this article, the limitations of the experiment, and future work.

## 2. Factors that Affect Sound Distance Perception

In order to achieve the sound effect that matches the subjective perception, researches on spatial sound perception have been conducted for a long time and mainly focus on distance perception and direction perception. This section mainly introduces factors that affect the spatial sound perception.

### 2.1. Intensity

In free sound field without reflection, sound pressure is the main factor that determines the subjective distance perception. Sound intensity decreases as the distance between sound source and listeners increases. The inverse-square law can be used to simulate sound distance in free sound field and can be expressed as in the following equation[13]:

(1) $$\begin{array}{c} \Delta P=20\text{ lg}\frac{p}{p_{0}}, \end{array}$$

where *P*_0_ is the sound pressure level (SPL) of reference distance, *P* is the SPL of simulating distance, and Δ*P* is the difference of SPL between the reference distance and simulating distance. But it should to be noted that the inverse-square law is only suitable for the free field sound and cannot be applied in room sound field (except for anechoic chamber).

There were many experiments on the effect of intensity on distance perception, and most of the experiments were focused on distance perception in free sound field [14]. So, the experimental environment was almost outdoor grass or anechoic chamber, which is approximate free sound field, and can simulate sound distance according to the inverse-square law.

In 1909, Gamble [15] proposed that in free sound field, intensity is one of the most important factors that affect sound distance perception, and the difference threshold of perceptual distance can be calculated by the inverse-square law. But Paul D. Coleman [16, 17] had another viewpoint. He proposed that the application of inverse-square or the 1/*R*^2^ theory in the inverse-square law is wrong because the relationship between distance perception and sound pressure is 1/*R*, and the relationship between distance perception and sound power is 1/*R*^2^. Since dB is used in SPL, it can also be used in proportional relationships of distance perception. Since measurements of loudness difference threshold were rough in early experiments, detailed data cannot be found in Gamble's experiment, so there was not sufficient data to support the conclusion about the relationship between distance and intensity.

Mershon and King [18] verified the affection of SPL to distance perception of sound. They carried experiments in an anechoic chamber, and there was no light at all. 80 Psychology students participated in the experiments, and their hearing loss was less than 15 dB during 1k∼8 kHz. All of them knew nothing about the experiment. Sound signal was 5s white noise which was generated by B&K noise generator. Loudspeakers were placed in the midline of listener's ears with the same height of ears (1.2 m). The distances between loudspeakers and listeners were 2.74 m and 5.49 m. The playing and stop time were controlled by an automatic electronic timer. There were three SPL, 45 dB, 55 dB, and 60 dB, which were all measured by B&K spectrum analyzer with A weighted at the head position. The experiment was divided into two groups. The further loudspeaker was played to the listeners in the first group, and the nearer speaker was played to listeners in the second group. Half of the listeners in each group listened the louder signals while the others listened the lower signals. Listeners can write down the sound source distance they perceived in any unit (inch, foot, or meter) or their combination. The result showed that intensity is a relative factor in egocentric distance perception but not the absolute distance, and sound source distance is typically overestimated for near distances and underestimated for far distances. Gardner [19], Von Békésy [20], and Zahorik came to the same conclusion.

In order to find out the effect of intensity on sound distance perception, factors that affect sound perception were divided into two parts according to the types of speech patterns in Brungart and Scott's experiments [21]. They were production level and presentation level. There were three types in the production level, whispering, conversation, and shouting. Presentation level was the signal intensity on the output side of headphone or speakers. The three types of speech were recorded, respectively, and the intensity was changed according to the inverse-square law. Speech contents were “Don't ask me to carry an oily rag like that,” “Over here,” “Threat,” and “Warming.” Experiments were carried out in a large playground, and subjects sat on chairs which were in the middle of the playground. There were nine signs marked a serial numbers (1∼9) at a distance 0.25 m, 0.5 m, 1m, 2m, 4m, 8m, 16m, 32m, and 64m from subjects, respectively. Subjects were asked to estimate the position of the speech source according to the spatial visual information of those signs in the playground and wrote down the sign number. The results showed that both production level and presentation level change as the distance perception changes. In free sound field, whispering cannot be estimated, and perceived distance of conversation and shouting increases as the sound source distance increases. For those speech with low SPL (less than 66 dB at 1m), distance doubles when the production level increases by 15 dB. If the production level of speech is low, it affects the distance perception only when the presentation level is more than 72 dB. If the production level of speech is high, perceived distance doubles when the production level increases by 8 dB or the presentation level decreased by 12 dB.

### 2.2. Direct-to-Reverberant Energy Ratio

In the environment with reflections, sound source location is affected by the acoustic ratio of direct and reverberation sound which is dependent on the geometric parameters of the room. In indoor environment, direct-to-reverberant energy ratio is important to sound source location, but there is also reverberation in some outdoor environment, which is also a factor that affects distance perception [22, 23].

Thrulow [24] found that direct sound energy decreases and reflected energy increases as the sound source moves away from the listener. Reverberation is an absolute factor that affects sound distance perception. In a natural indoor environment, listeners can estimate sound source distance by direct sound and reflected sound energy in the first time they hear the sound [25]. At a nearer distance, direct sound arrives at listener's ears first. When the sound is far from listeners, reflected sound plays the main role in distance perception, while direct sound still obeys the inverse-square law. But Michael Schutte et al. supposed that the estimation of subjects to reverberation did not depend on visual cues [26].

### 2.3. Spectrum

The spectrum of sound we heard is different from what it should be because of the absorption of the air. The absorption of frequency by air is different. High frequency is absorbed more significantly by the air, so the spectrum of sound changes significantly during high-frequency attenuation, especially when the sound has a broad bandwidth and transmits a long distance. Spectrum of sound contains information on the sound source distance [27]. In addition, in the environment with reflections, the spectrum of sound that was received by listeners is also affected by the acoustic characteristics of the reflectors. Different reflectors have different effects on frequency. In a room with reflections, reflected energy increases as the distance increases, which leads the spectrum of sound received by listeners to change.

The change of spectrum which is determined by the loss of sound energy is the dependent variable of frequency and distance. The higher-frequency sounds do not travel as far as lower frequency because the loss in energy of higher frequency is more than lower frequency. If the sound travels far enough, listeners cannot feel the high frequency, so the frequency spectrum includes distance information. Coleman found that spectrum has dual effects on distance perception in his experiment. When he analyzed the physical characteristic of the sound signal, he found that high frequency attenuates faster than low frequency for a complex sound signal, and the conclusion was established both for near distance (less than 1m) and far distance (more than 3m). Based on the conclusion, he carried experiment to verify that the attenuation of high frequency makes the nearer sound perceive much closer and the further sound perceive much further.

### 2.4. Binaural Differences

For far-field plane waves, binaural intensity difference and binaural time difference are irrelevant to sound source distance, while for near-field condition, binaural intensity difference and binaural time difference are relevant to sound source distance. The differences are greatest in binaural axis and smallest in the vertical plane of the binaural axis [28, 29]. Besides, in the near field, because of the diffraction caused by head and pinna, sound spectrum changes as the distance between sound source and listeners changes. Head-related transform function (HRTF) is used to represent the diffraction effect of head and pinna [30–32].

### 2.5. Nonacoustic Factors

Although acoustic factors are the most important factors that affect sound distance perception, some nonacoustic factors also have affection on distance perception [33].

The familiarity with the sound source by the listener is another important factor that affects sound distance perception [34]. Based on the familiarity with the sound, listeners can estimate some factors that are relevant to sound distance perception, such as intensity and spectrum. Distance estimation is more accurate when the sound source is a familiar sound than unfamiliar ones [35], and the accuracy will improve as the repetition increases.

Brungart and Scott [36] found that the sound types (speech or other sound signals) have little effect on distance perception in their experiments about production level and presentation level, but the familiarity with sound source and intensity may affect sound distance perception. Coleman found the importance of familiarity with sound source in sound distance experiment and the result showed that distance perception of speech is significantly different from white noise by subjects [37].

In theory, HRTF has effect on distance perception too, but Pavel Zahorik found that individualized HRTF did not perform better than non-individualized HRTF on distance perception [38]. There were 6 subjects participating in the experiment to estimate the distance. The result showed that there is no significant difference between individualized HRTF and nonindividualized HRTF in distance location. The accuracy of distance location by ears did not decrease as the application of nonindividualized HRTF.

Sound source or receivers' movement are also important factors that affect distance perception [39–45]. Different from those experiments carried out in static condition, Ashmead carried a subjective evaluation experiment to evaluate distance perception in movement condition which was mentioned before. The experiment was carried out in a large outdoor space, and sound signal which was 1500 ms white noise was played back by loudspeakers. Subjects were blindfolded in the experiment, and they were asked to walk to the sound source position they perceived according to the sound signals they heard. The subjects had two listening conditions. One was in a stationary state, that is to say, the subjects stayed still when they were listening and walked to the source position they perceived after the sound played completely. The other one was in the state of motion, that is to say, the subjects walked to the sound source position as they hear the sound. The experiment measured whether the subjects' movement is helpful to distance perception. The result showed that sound location is more accurate and consistent when subjects are under moving condition.

Among those nonacoustic factors, visual information has a great effect on sound perception [46, 47]. The visible objects can attract and capture sound source perceptual position, which is called “ventriloquism effect,” especially for directional localization. “Ventriloquism effect” can offset some angles for auditory events towards the visual target. For distance localization, “ventriloquism effect” also works [48]. Many researches have come to the same conclusion that visual targets have a capturing and controlling effect on auditory events in a large distance range. When the auditory source is further than the visual source, the perceived auditory distance is less than the actual distance [49]. In all, the visual target can improve the accuracy of sound localization and decrease the variance and fluctuation of distance estimation especially in the existence of more than one visual target.

There are two types of visual stimuli in sound perception experiments. One is the visual stimuli in an actual space, such as glitter of light source and visible or invisible sound source, and the other one is visual display on-screen, including intensity, pure color, and splash. Many experiments focused on direction perception, and little experiment focused on distance perception.

Anyway, most of the experiments on distance perception focused on the effect of intensity, and intensity is the most important factor that affects auditory distance perception. But systematic measurement of attenuating SPL that matches the visual sound source in virtual environment can rarely be seen. Two experiments were conducted in this paper. The first experiment was carried out to compare the auditory distance perception of subjects in the existence and absence of visual sound source. Based on the result of the first experiment, the second experiment was conducted to measure the best attenuating SPL of the initial sound signal that made the subjects perceive the best audiovisual distance in the existence of virtual visual sound source at a distance of 4m, 6m, 8m, 10m, and 12m.

## 3. Comparison of Auditory Distance Perception in the Existence and Absence of Visual Cue

### 3.1. Materials

Materials included visual signals and audio signals. Visual signals were virtual images which were series of photos taken in a large open playground. Audio stimulus was speech that was recorded in a recording room.

#### 3.1.1. Collection and Processing of Visual Signals

Visual signals were a series of photos taken in a large open playground, and the main subject in the image was a loudspeaker with a stand. The distances between the loudspeaker and camera were from 2m to 32m with an interval of 2m. There were only grassland, the loudspeaker, and stand in the image by erasing the redundant information after postprocessing. The loudspeaker in the picture was the virtual visual sound source in the experiment. A more detailed explanation of the collection and postprocessing of the pictures is shown below.

- The camera retains its place in taking pictures with a height of 1.2 m
- Standard lens is used
- No zooming
- The lens faces the loudspeaker with no angle of tilt
- The loudspeaker retains its height of 1.2 m when it moves
- The loudspeaker moves along a straight line, and its motion track is not curved
- The pictures were postprocessed to erase the fence of the playground and the buildings in far distance and replaced with the sky color

#### 3.1.2. Recording and Processing of Audio Signals

**Figure 1:**
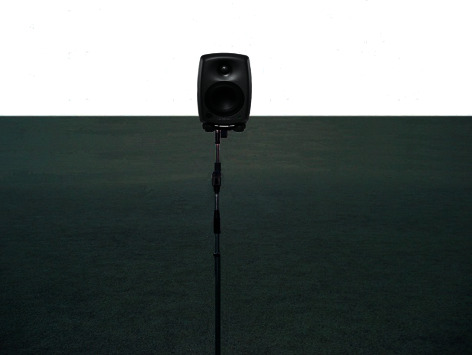
The picture of virtual sound source.

Audio stimulus is speech that is recorded in a recording room. The speaker is a male who has been taking pronunciation training. Speech content is “Hey, I'm here!” in Chinese. The reason for choosing the speech content is that people are familiar with the content, and it has a potential hint for distance estimation as the virtual sound source moves. The SPL is 66 dB which is measured 10 cm in front of the mouth of the speaker (Figure 1).

The recorded speech is taken as the initial signal to match the virtual visual sound source on the screen at a distance of 2m. Sound signals at other distances were simulated according to the inverse-square law based on the initial signal because the open playground is a typical free acoustic field. The variation of distance between sound source and receiver obeys the inverse-square law, which means SPL decreases 6 dB as distance doubles. Then, taking the sound signal corresponding to the virtual visual sound source at 2m as the initial signal, the sound signals of the virtual sound source at 4m, 6m, 8m, 10m, 12m, 14m, 16m, 18m, 20m, 22m, 24m, 26m, 28m, and 32m were simulated, and the attenuating SPL for each sound signal is shown in Table 1.

#### 3.1.3. Experimental Signal Synthesis

The experimental signals included two groups. In the first group, the virtual sound source can be seen when the sound signals were played back to the listeners, and there was only grassland without the virtual visual sound source on the screen in the second group. Taking the synthesis of sound signals with virtual sound source can be seen as an example to show how the experimental signals are synthesized.

- First of all, play the pictures with virtual visual sound sources at a distance of 2m and 32m and their corresponding sound signals, respectively, as the reference signals
- After the reference signals, play the picture with virtual visual sound source at distance of 4m and its corresponding sound signal that attenuated SPL according to Table 1 based on the initial sound signal
- Sound signals with virtual visual sound source at other distances under test were made in the same way
- All the signals were synthesized to a group of video-audio signals in a random order

The synthesis of experimental signals with the sound source invisible was in the same way, but there was only grassland in the picture when the sound signals under test were played back.

### 3.2. Subjects

There were 15 subjects who participated in the experiment. All the subjects were graduates in university who had basic acoustic knowledge and had experienced subjective experiments. All the subjects reported having normal hearing.

### 3.3. Device Connection

#### 3.3.1. Video Devices

**Figure 2:**
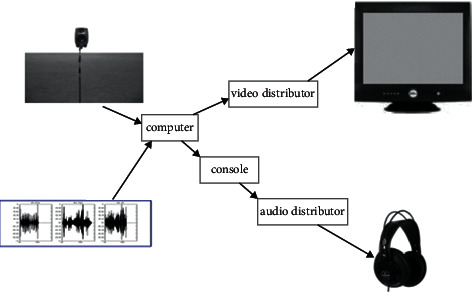
The schematic of the device connection in the experiment.

Video devices were 10-inch flat-panel monitors (Dell M782), and the parameters of all the monitors like luminance, hue, and saturation were adjusted to the same values. Video signals were transmitted to the monitors through video distributors (HYTVGAD0104) from the main computer at the meantime, and there was no time delay among the monitors.  Figure 2 shows the schematic of the device connection in the experiment.

#### 3.3.2. Audio Devices

Sound signals were transmitted through a mini console (DEITY MX-8S mini) and an audio distributor (HYT-DAV0108) from the main computer to headphones (AKG 240DF), and the volume was controlled by the audio distributor. The SPL was 70 dB at the output of the headphone measured by a tiny microphone installed in a dummy head, and subjects could not operate the monitor devices.

### 3.4. Procedure

- Each subject was distributed a monitor and a headphone.
- The subjects were asked to watch the monitor directly without any head tilting.
- The distances between the seat and the monitor of each subject were the same and remained constant.
- The experiment with no visual cues was carried out first. Subjects estimated the distance of the sound signals under test without virtual sound source in the picture. They wrote down the distance of the sound signals they perceived in meters by taking the pictures and sound signals of virtual sound sources at distance of 2m and 32m as references.
- Subjects were asked to take ten minutes' break after they finished the first experiment.
- After the ten minutes' break, the second group of experiment was carried out. When the subjects listened to the sound signals under test, there were also corresponding virtual visual sound sources on the screen. The subjects can estimate the distances of the sound signals not only according to the reference sound signals but also virtual visual sound sources corresponding to the sound signals under test. They wrote down the distances of the sound signals in meters.

## 4. Results

**Figure 3:**
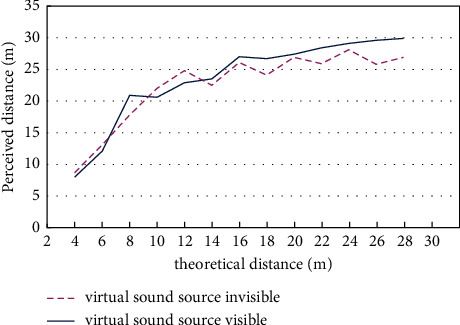
Distance perception in the existence and absence of virtual sound source on the screen.

Perceived distances of all subjects were put into the computer, and the results were calculated by averaging the data of the subjects for every measured distance. Distance perception in the existence and absence of virtual sound source on the screen was shown in Table 2 and Figure 3.

Figure 3 showed that the trends of sound distance perception in the existence and absence of virtual sound source are almost the same. Perceived distance is larger than the theoretical distance in measurement ranges and also larger than those results of distance perception measured in actual environments [50, 51].

Although the trends of distance perception are almost the same in the existence and absence of virtual visual sound source on the screen, when measured distance is far, perceived distance in the existence of virtual sound source on the screen is a little larger than those results measured in the absence of virtual sound source on the screen, and the curve is less fluctuant in the existence of virtual sound source. Visual cue can increase the stability of distance perception, and the affection of visual cue on sound distance perception increases as the perceived distance increases.

## 5. Experiment on Best Distance Perception

### 5.1. Method

Paired comparison method which is a basic method for measuring rank order was used in the experiment. The basic principle of paired comparison method is that psychological value is a random variable conforming to normal distribution. Pair up all the stimuli to be compared and present them in pairs. Subjects are asked to compare the stimuli for a feature and judge which one is more obvious. Each stimulus needs to be compared separately to the others. Suppose *n* is the total number of stimuli, then the number of pairs is *n*(*n* − 1)/2. Finally, an ordinal scale can be made by placing them in order of the size of their respective percentages which are more obvious than other stimuli [52].

### 5.2. Experimental Signals

Postprocessing of video and recording of audio signals were the same as the procedures described in section 3.1. But postprocessing of audio signals and synthesis of experimental signals were more complex because the experimental method was different from the last experiment.

#### 5.2.1. Postprocessing of Audio Signals

Taking the recorded speech as the initial sound signal which corresponds to the virtual visual sound source at a distance of 2m, simulated sound signals match the virtual visual sound sources at 4m, 6m, 8m, and 12m on the screen by attenuating the SPL of the initial sound signal according to the inverse-square law, respectively. The attenuated signals were called reference signals, and the theoretical attenuation is shown in Table 3.

Comparison signals were obtained by attenuating the SPL of the reference sound signals. Because paired comparison method was chosen in the experiment, pilot experiment was carried out to set reasonable interval of comparison signals to decrease tests and ensure the effectiveness of the experiment. Attenuating SPL of comparison signals for each reference signal of the virtual sound source is shown in Table 4.

#### 5.2.2. Synthesis of Experimental Signals

- Taking the production of reference signal corresponding to virtual visual sound source at 4m as an example, two comparison signals of the reference signal are paired corresponding to virtual sound source at 4m in pairs
- The initial sound signal and its corresponding virtual visual sound source were played first before each pair of comparison signals were played
- Each pair of comparison signals was played accompanied with the virtual visual sound source at distance of 4m on the screen
- The syntheses of other pairs of comparison signals of reference signals that correspond to virtual sound source at a distance of 6m, 8m, and 12m followed the same steps
- When all the comparison signals had been combined in pairs, arranged all the pairs of comparison signals randomly

### 5.3. Procedure

#### 5.3.1. Connection of Video and Audio Devices

Connection of video and audio devices was the same as those described in section 2.3.

#### 5.3.2. Description of Experiment

- Each subject was distributed a monitor and a headphone.
- The subjects were asked to watch the monitor directly without any head tilting.
- The distance between the subject's head and the monitor was 50 cm.
- The subjects were asked to select the option that makes them obtain the best distance perception from the two comparison signals in a pair based on the reference sound signal and its corresponding virtual sound source on the screen and draw “✓” in the corresponding table.
- The experiment was divided into three parts and each part of the experiment needed 50 minutes to be finished. There were 10 minutes between two parts to allow subjects to take a break.

### 5.4. Results

#### 5.4.1. Numeralization of Data

Before data processing, data need to be numerically processed because subjects marked “✓” not numbers in the blank. The rule of numeralization is that in each pair of comparison signals, the signal which is marked “✓” (the signal that subject had better distance perception) is given a value of 1, and the other one is given a value of 0.  Table 5 showed the rule of numeralization.

#### 5.4.2. Reliability Test

Reliability test is used to test the reliability and authenticity of subjects' choices because the subjects cannot keep focusing on making choices. Unreliable data can be found and deleted after the reliability test.

Reliability test is performed by calculating the consistency coefficient which is computed by testing the same signal twice for the same subject. If the judgments of a subject in two tests come to a high consistency, then the reliability of the data is good and can be used in data processing. If the consistency of the two test results for a subject is poor, the data must be deleted.

Computational formula of the consistency coefficient is shown in the following equation:

(2) $$\begin{array}{c} r_{\text{tt}}=\frac{\text{sum}\left|A\left(:,n\right)-B\left(:,n\right)\right|}{n}, \end{array}$$

where *A* and *B* are matrices with the same number of rows and columns. *A* represent data of the first experiment, while *B* represents the data of the second experiment, and *n* is the number of pairs of comparison signals. Consistency coefficient is a number between 0 and 1.

Each signal in a pair of comparison signals has one time to be played first, so each pair of comparison signals need to be played twice to ensure each signal has one chance to be played first. XOR is used to calculate the consistency coefficient of all the subjects' data. Consistency coefficient of the experiment is shown in Table 6.

Table 6 showed that the fluctuation range of the consistency coefficient is very large. Subjects' data whose consistency coefficient was more than 60% were valid, and the consistency coefficient that was less than 60% was deleted to ensure the experiment result was effective.

#### 5.4.3. Results

**Figure 4:**
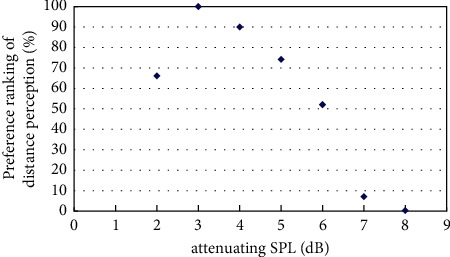
Preference of attenuating SPL in the existence of virtual visual sound source at distance of 4m.

**Figure 5:**
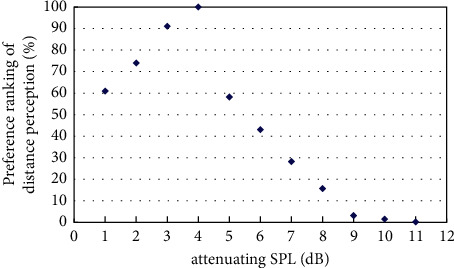
Preference of attenuating SPL in the existence of virtual visual sound source at distance of 6m.

**Figure 6:**
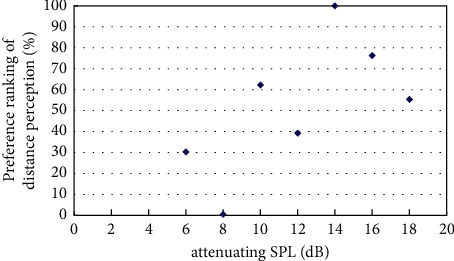
Preference of attenuating SPL in the existence of virtual visual sound source at distance of 8m.

**Figure 7:**
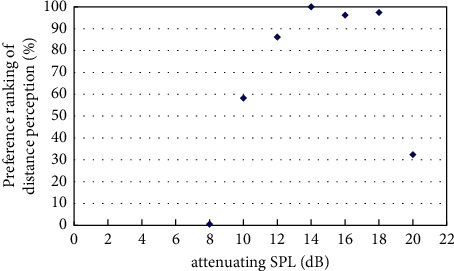
Preference of attenuating SPL in the existence of virtual visual sound source at distance of 10m.

**Figure 8:**
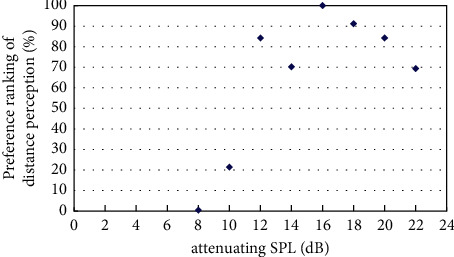
Preference of attenuating SPL in the existence of virtual visual sound source at distance of 12m.

The experiment on best distance perception measured the best sound distance perception with the existence of virtual visual sound source at a distance of 4m, 6m, 8m, and 12m based on the initial virtual sound source at a distance of 2m and its corresponding sound signal by pair comparison method. The results of the experiment were obtained by classifyingand calculatingthe valid data, andthe resultsare shown in Figures 4–8.

Figure 4 showed the preference ranking of sound distance perception when the virtual visual sound source was 4m and the attenuating SPL was between 2 and 8 dB. And when the SPL of the initial sound was attenuated to 3 dB, subjects can obtain the best audiovisual distance perception.

Figure 5 showed the preference ranking of sound distance perception when the virtual visual sound source was 6m and the attenuating SPL was between 6 and 16 dB. And when the SPL of the initial sound was attenuated to 9 dB, subjects can obtain the best audiovisual distance perception.

Figure 6 showed the preference ranking of sound distance perception when the virtual visual sound source was 8m and the attenuating SPL was between 6 and 18 dB. And when the SPL of the initial sound was attenuated to 14 dB, subjects can obtain the best audiovisual distance perception.

Figure 7 showed the preference ranking of sound distance perception when the virtual visual sound source was 10m and the attenuating SPL was between 8 and 20 dB. And when the SPL of the initial sound was attenuated to 14 dB, subjects can obtain the best audiovisual distance perception.

Figure 8 showed the preference ranking of sound distance perception when the virtual sound source was 12m and the attenuating of SPL was between 8 and 22 dB. And when the SPL of the initial sound was attenuated to 16 dB, subjects can obtain the best audiovisual distance perception.

## 6. Discussion

Experiment on best distance perception was carried out to measure the SPL attenuation of best distance perception. The subjective ranking of the simulated sound signals followed the extreme law for the same virtual visual sound source. When the SPL of the initial sound was attenuated to 3 dB, subjects can obtain the best distance perception with the existence of virtual sound source at a distance of 4m. When the SPL of the initial sound was attenuated to 9 dB, subjects can obtain the best distance perception with the existence of virtual sound source at a distance of 6m. When the SPL of the initial sound was attenuated to 14 dB, subjects can obtain the best distance perception with the existence of virtual sound source at a distance of 8m. When the SPL of the initial sound was attenuated to 16 dB, subjects can obtain the best distance perception with the existence of virtual sound source at a distance of 12m.

**Figure 9:**
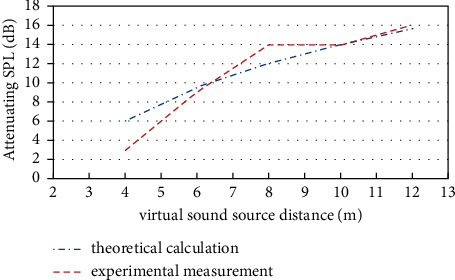
Attenuating SPL of experiment measurement and theoretical calculation.

The attenuating SPL obtained by the experiment of best distance perception is different from theoretical calculation according to the inverse-square law for the virtual visual sound source at the same position. The difference between the two methods is shown in Figure 9.

Figure 9 showed that there is a little difference between theoretical calculation and experimental measurement. When sound source distance is less than 6m, attenuating SPL of experimental measurement is less than theoretical calculation, and when sound source distance is about 6m, the two values would be approximately the same. When virtual visual sound source distance is between 6m and 8m, attenuating SPL of experimental measurement is more than theoretical calculation, and when sound source distance is more than 10m, the two values are the same again. But the experiment cannot show the difference when the virtual sound source is more than 12m, and it should be done in future work.

Besides, attenuating the SPL of the initial signal in the existence of virtual sound source at 8m is the same as the value of virtual sound source at 10m. The reason may be that visual perception of the virtual visual sound source at a distance of 8m and 10m is similar and the interval of attenuating SPL of the test sound signal is not small enough, and further research should be done to distinguish the auditory distance perception in the existence of virtual sound source at 8m and 10m.

## 7. Conclusions, Limitations, and Future Work

In this paper, the experiment on the effect of visual cue on auditory perception was conducted to investigate the effect of virtual visual cue on sound distance perception. The result showed that there is no obvious difference between the estimation of auditory distance perception in the existence and absence of virtual sound source on the screen, but the perception of auditory distance is less fluctuating when there is a virtual sound source on the screen. And another psychoacoustic experiment on best distance perception was carried out to measure the optimal attenuating SPL of the initial sound signal that makes the subjects obtain the best audiovisual distance perception when the virtual visual sound source was at a distance of 4m, 6m, 8m, 10m, and 12m, and draw the preference ranking of attenuating SPL of the initial signal in the existence of virtual sound source at a distance of 4m, 6m, 8m, 10m, and 12m, respectively.

The main limitation in this work is the chosen virtual sound source distance is not far enough, and the difference between the attenuating SPL obtained by the two methods cannot be found when the virtual sound source is more than 12m. In the future work, we will investigate the best distance perception at far distance and optimize the auditory distance perception experiment to achieve the best audiovisual perception.

## Figures and Tables

**Table 1:** Theoretical attenuating SPL of the distance of virtual sound source.

Distance of virtual sound source (m)	4	6	8	10	12	14	16	18	20	22	24	26	28	32
Attenuating SPL (dB)	6	9.5	12	14	15.6	16.9	18	19.1	20	20.8	21.6	22.3	22.9	24.1

**Table 2:** Distance perception in the existence and absence of virtual visual sound source on the screen.

Theoretical distance (m)	Perceived distance (m)	
	Virtual sound source visible	Virtual sound source invisible
4	8	8.7
6	12.1	13.1
8	20.9	17.8
10	20.6	22
12	22.9	24.8
14	23.5	22.5
16	27	26.1
18	26.7	24.1
20	27.4	26.9
22	28.4	25.9
24	29.1	28.1
26	29.6	25.8
28	29.9	26.9

**Table 3:** Theoretical attenuation of reference sound signals.

Virtual sound source distance (m)	4	6	8	10	12
Attenuating SPL (dB)	6	9.5	12	14	15.6

**Table 4:** SPL attenuation of comparison signals for each reference signal of virtual sound source.

Reference signal of virtual sound source (m)	Attenuating SPL (dB)							
4	2	3	4	5	6	7	8	N/A
6	6	7	8	9	10	12	14	16
8	6	8	10	12	14	16	18	N/A
10	8	10	12	14	16	18	20	N/A
12	8	10	12	14	16	18	20	22

**Table 5:** Numeralization of experiment data.

Value	Rule of numeralization
1	Signal that was marked “✓”
0	Signal that subjects did not choose

**Table 6:** Results of reliability test.

Virtual sound source at 4m	Subject No.	01	02	03	04	05	06	07	08	09
	Consistency coefficient	57%	52%	61%	14%	52%	57%	62%	62%	38%
	Subject no.	10	11	12	13	14	15	16	17	N/A
	Consistency coefficient	67%	81%	86%	67%	76%	62%	57%	67%	N/A

Virtual sound source at 6m	Subject no.	01	02	03	04	05	06	07	08	09
	Consistency coefficient	35%	58%	58%	19%	54%	65%	85%	58%	65%
	Subject no.	10	11	12	13	14	15	16	17	N/A
	Consistency coefficient	62%	73%	92%	77%	73%	73%	62%	42%	N/A

Virtual sound source at 8m	Subject no.	01	02	03	04	05	06	07	08	09
	Consistency coefficient	57%	57%	76%	14%	62%	62%	67%	52%	67%
	Subject no.	10	11	12	13	14	15	16	17	N/A
	Consistency coefficient	71%	95%	81%	62%	71%	67%	52%	62%	N/A

Virtual sound source at 12m	Subject no.	01	02	03	04	05	06	07	08	09
	Consistency coefficient	61%	39%	64%	46%	75%	82%	57%	82%	64%
	Subject no.	10	11	12	13	14	15	16	17	N/A
	Consistency coefficient	54%	93%	43%	43%	64%	61%	54%	75%	N/A

## Data Availability

The data used to support the findings of this study are included within the article.

## References

[1] Li X., Deng X., Xu H. (2022). Interactive cultural communication effect in VR space of intelligent mobile communication network. *Wireless Communications and Mobile Computing*.

[2] Yin W. (2022). An artificial intelligent virtual reality interactive model for distance education. *Journal of Mathematics*.

[3] Yao J., Liu G., Ying C. Image quality assessment based on the visual perception of image contents.

[4] Ambalathankandy P., Shimada T., Takamaeda S., Motomura M., Asai T., Ikebe M. Analysis of smoothed LHE methods for processing images with optical illusions.

[5] Sakai Y., Katsura S. Modeling and control of sound system for sound field reproduction.

[6] Kamado N., Saruwatari H., Shikano K. Robust sound field reproduction integrating multi-point sound field control and wave field synthesis.

[7] Mróz B., Kostek B. (2022). *Pursuing Listeners’ Perceptual Response in Audio-Visual Interactions–Headphones vs Loudspeakers: A Case Study*.

[8] Valzolgher C., Giovanelli E., Sorio R., Rabini G., Pavani F. (2022). Can visual capture of sound separate auditory streams?. *Experimental Brain Research*.

[9] Zhao J., Zheng X., Ritz C., Jang D. (2022). Interpolating the directional room impulse response for dynamic spatial audio reproduction. *Applied Sciences*.

[10] Zhang Q. (1999). *Research on Generalized Virtual Acoustic Environment(in Chinese)*.

[11] Kleiner M., Dalenback B., Svensson P. (1993). Auralization – an overview. *J.Aud.Eng. Soc*.

[12] Neidhardt A., Schneiderwind C., Klein F. (2022). Perceptual matching of room Acoustics for auditory augmented reality in small rooms-literature review and theoretical framework. *Trends in Hearing*.

[13] Coleman P. D. (1963). An analysis of cues to auditory depth perception in free space. *Psychological Bulletin*.

[14] Rao P. (2001). Applying perceptual distance to the discrimination of sounds. *Proceedings of the National Conference on Communications, I.I.T. Kanpur, Kanpur, Uttar Pradesh, January 2001*.

[15] Gamble E. A. (1909). Minor studies from the psychological laboratory of Wellesley College: intensity as a criterion in estimating the distance of sounds. *Psychological Review*.

[16] Hartmann W. M. Localization of a source of sound in a room.

[17] Schuck P. L., Olive S. E., Ryan J. G., Toole F. E. Perception of perceived sound in rooms:some results of the athena project.

[18] Mershon D. H., King L. E. (1975). Intensity and reverberation as factors in the auditory perception of egocentric distance. *Perception & Psychophysics*.

[19] Gardner M. B. (1969). Distance estimation of 0° or apparent 0°‐oriented speech signals in anechoic space. *Journal of the Acoustical Society of America*.

[20] Békésy G. V. (1949). The moon illusion and other similar auditory phenomena. *American Journal of Psychology*.

[21] Brungart D. S., Scott K. (2001). The effects of production and presentation level on the auditory distance perception of speech. *Journal of the Acoustical Society of America*.

[22] Richards D. G., Wiley R. H. (1980). Reverberations and amplitude fluctuations in the propagation of sound in a forest: implications for animal communication. *The American Naturalist*.

[23] Makous J. C., Middlebrooks J. C. (1990). Two-dimensional sound localization by human listeners. *Journal of the Acoustical Society of America*.

[24] Thurlow H. J. (1971). Illness in relation to life situation and sick-role tendency. *Journal of Psychosomatic Research*.

[25] Mershon D. H., Bowers J. N. (1979). Absolute and relative cues for the auditory perception of egocentric distance. *Perception*.

[26] Schutte M., Ewert S. D., Wiegrebe L. (2019). The percept of reverberation is not affected by visual room impression in virtual environments. *Journal of the Acoustical Society of America*.

[27] Little A. D., Mershon D. H., Cox P. H. (1992). Spectral content as a cue to perceived auditory distance. *Perception*.

[28] Mills A. W. (1958). On the minimum audible angle. *Journal of the Acoustical Society of America*.

[29] Kelly J. W., Loomis J. M., Beall A. C. (2004). Judgments of exocentric direction in large-scale space. *Perception*.

[30] Duda R. O., Martens W. L. (1998). Range dependence of the response of a spherical head model. *Journal of the Acoustical Society of America*.

[31] Brungart D. S., Rabinowitz W. M. (1999). Auditory localization of nearby sources. Head-related transfer functions. *Journal of the Acoustical Society of America*.

[32] Jack C. E., Thurlow W. R. (1973). Effects of degree of visual association and angle of displacement on the “ventriloquism” effect. *Perceptual & Motor Skills*.

[33] Begault D. R. Auditory and non-auditory factors that potentially influence virtual acoustic imagery.

[34] Zahorik P. (2000). Distance localization using nonindividualized head‐related transfer functions. *Journal of the Acoustical Society of America*.

[35] Coleman P. D. (1962). Failure to localize the source distance of an unfamiliar sound. *Journal of the Acoustical Society of America*.

[36] Hollier M. P., Cosier G. (1996). Assessing human perception. *BT Technology Journal*.

[37] Cabrera D., Gilfillan D. Auditory distance perception of speech in the presence of noise.

[38] Zahorik P. (2002). Assessing auditory distance perception using virtual acoustics. *Journal of the Acoustical Society of America*.

[39] Ashmead D. H., Davis D. L., Northington A. (1995). Contribution of listeners’ approaching motion to auditory distance perception. *Journal of Experimental Psychology: Human Perception and Performance*.

[40] Thurlow W. R., Kerr T. P. (1970). Effect of a moving visual environment on localization of sound. *American Journal of Psychology*.

[41] Larsson P., Vastfjall P., Kleiner M. Perception of self motion and presence in auditory virtual environments.

[42] Altman J. A., Romanov V. P., Pavlov I. P. (1988). Psychophysical characteristics of the auditory image movement perception during dichotic stimulation. *International Journal of Neuroscience*.

[43] Harris J. D., Sergeant R. L. (1971). Monaural/binaural minimum audible angles for a moving sound source. *Journal of Speech & Hearing Research*.

[44] Dapelo R., Macelloni S. A real time algorithm for stereophonic-multi-channel locatization of moving sound sources.

[45] Carlile S., Best V. (2002). Discrimination of sound source velocity in human listeners. *Journal of the Acoustical Society of America*.

[46] Anderson P. W., Zahorik P. (2011). Auditory and visual distance estimation. *Journal of the Acoustical Society of America*.

[47] Postma B. N. J., Katz B. F. G. (2017). The influence of visual distance on the room-acoustic experience of auralizations. *Journal of the Acoustical Society of America*.

[48] Paquier M., Cote N., Devillers F., Koehl V. (2016). Interaction between auditory and visual perceptions on distance estimations in a virtual environment. *Applied Acoustics*.

[49] Kolarik A. J., Cirstea S., Pardhan S., Moore B. (2013). An assessment of virtual auditory distance judgments among blind and sighted listeners. *Journal of the Acoustical Society of America*.

[50] Zahorik P. Auditory display of sound source distance.

[51] Pellegrini R. S. Perception-based design of virtual rooms for sound reproduction.

[52] Meng Z. (2007). *Psychological Test Methods for Sound Quality Evaluation and Hearing Research*.

